# Temperature‐dependent alterations in the proteome of the emergent fish pathogen *Edwardsiella piscicida*

**DOI:** 10.1111/jfd.14017

**Published:** 2024-09-20

**Authors:** Kim L. Jacobsen, Matt Griffin, Brett S. Phinney, Michelle Salemi, Zeinab Yazdi, Sujita Balami, Caitlin E. Older, Esteban Soto

**Affiliations:** ^1^ Department of Medicine and Epidemiology, School of Veterinary Medicine University of California Davis California USA; ^2^ Department of Pathobiology and Population Medicine, College of Veterinary Medicine, Thad Cochran National Warmwater Aquaculture Center, Delta Research and Extension Center Mississippi State University Stoneville Mississippi USA; ^3^ Proteomics Core Facility University of California Davis California USA; ^4^ Warmwater Aquaculture Research Unit, Agricultural Research Service U.S. Department of Aquaculture Stoneville Mississippi USA

**Keywords:** aquaculture, *Edwardsiella piscicida*, edwardsiellosis, proteomics, virulence factor

## Abstract

*Edwardsiella piscicida* is an emerging bacterial pathogen and the aetiological agent of edwardsiellosis among cultured and wild fish species globally. The increased frequency of outbreaks of this Gram‐negative, facultative intracellular pathogen pose not only a threat to the aquaculture industry but also a possible foodborne/waterborne public health risk due to the ill‐defined zoonotic potential. Thus, understanding the role of temperature on the virulence of this emerging pathogen is essential for comprehending the pathogenesis of piscine edwardsiellosis in the context of current warming trends associated with climate change, as well as providing insight into its zoonotic potential. In this study, significant temperature‐dependent alterations in bacterial growth patterns were observed, with bacterial isolates grown at 17°C displaying higher peak growth sizes, extended lag times, and slower maximal growth rates than isolates grown at 27or 37°C. When *E. piscicida* isolates were grown at 37°C compared to 27 and 17°C, mass spectrometry analysis of the *E. piscicida* proteome revealed significant downregulation of crucial virulence proteins, such as Type VI secretion system proteins and flagellar proteins. Although in vivo models of infection are warranted, this in vitro data suggests possible temperature‐associated alterations in the virulence and pathogenic potential of *E. piscicida* in poikilotherms and homeotherms.

## 1 INTRODUCTION

*Edwardsiella piscicida* is a Gram‐negative, facultative anaerobic, facultative intracellular bacterium belonging to the family *Hafniaceae*. This bacterium is an emerging pathogen of both freshwater and marine fish species worldwide, including economically important cultured and sport fish species in the United States such as catfish (*Ictalurus* spp.), trout (*Oncorhynchus* spp.), and black bass (*Micropterus* spp.) (Griffin et al., 2014, 2019; Leung et al., 2019; Reichley et al., 2017). Fish infected with *E. piscicida* typically present with clinical signs of septicaemia, characterized by multifocal external and internal petechial haemorrhages, skin discolouration and ulceration, exophthalmia, and haemorrhagic ascites (Buján et al., 2018; Griffin et al., 2020). Historically, the genus *Edwardsiella* was comprised of three species: *E. tarda*, a bacterium with a wide host range, including both fish and mammals; *E. ictaluri*, the causative agent of enteric septicaemia of catfish and outbreaks of edwardsiellosis in tilapia; and *E. hoshinae*, which is primarily considered a commensal in birds and reptiles (Griffin et al., 2020). In 2012–2013, multilocus sequence analysis studies revealed the polyphyletic origins of *E. tarda* isolates collected from diverse host fish species (Abayneh et al., 2012; Griffin et al., 2013), leading to the taxonomic reorganization of *E. tarda* into three distinct species: *E. tarda* (Abayneh et al., 2013; Reichley et al., 2017), *E. piscicida* (Abayneh et al., 2013; Griffin et al., 2014; Reichley et al., 2017), and *E. anguillarum* (Reichley et al., 2017; Shao et al., 2015). Subsequent genotypic and phenotypic studies have further identified at least six distinct clades within the species *E. piscicida*, with experimentally demonstrated differences in host specificity and disease severity (Nguyen et al., 2021).

In the decade since its recognition as a discrete taxa, *E. piscicida* has been increasingly isolated from disease outbreaks and mass mortality events among wild and cultured fish species (Leung et al., 2019). Not only has there been increased frequency of isolation of *E. piscicida* from wild and cultured fish, but the host and geographic range have seemingly expanded (Griffin et al., 2019, 2020). The global emergence of *E. piscicida* not only threatens wild and cultured fish populations but could also pose a potential public health risk. There is currently no clear consensus in the literature on the capacity for *E. piscicida* to infect and cause disease in mammals, owing in large part to historical conflation with the closely related species *E. tarda* (Griffin et al., 2020). *Edwardsiella tarda* is known to infect a wide range of taxa, including fish, reptiles, and terrestrial endotherms (Griffin et al., 2020). In humans, infections with *E. tarda* are rare but can lead to significant and potentially fatal intestinal and/or extraintestinal disease (Hirai et al., 2015). Human infections with *E. tarda* are most commonly associated with exposure to contaminated water or consumption of infected animals (Hirai et al., 2015). Thus, the rise of *E. piscicida* among farmed fish populations could pose a growing foodborne and/or water‐borne disease risk. Accurate assessment of the zoonotic risk of this pathogen necessitates further investigation into the potential virulence of *E. piscicida* in a mammalian model of infection.

*Edwardsiella piscicida* relies on several crucial virulence factors to evade host immune responses, invade host cells, replicate intracellularly, and disseminate throughout host tissues. Most notable among the virulence factors implicated in the pathogenesis of *E. piscicida* are the type III (T3SS) and type VI (T6SS) secretion systems, which are essential for successful *E. piscicida* infection (Leung et al., 2019; Zheng & Leung, 2007). These secretion systems are comprised of protein apparatuses that directly inject cytotoxic effectors into host cells to facilitate bacterial invasion and intracellular replication (Leung et al., 2019; Zheng & Leung, 2007). Other virulence factors associated with *E. piscicida* pathogenesis include iron uptake regulators (Swain et al., 2020), oxidative stress factors (Gao et al., 2016; He et al., 2023), haemolysin (Buján et al., 2018; Wang et al., 2010), translocation and assembly modules (TAM) (Li et al., 2020), two‐component systems (Chakraborty et al., 2011; Zheng et al., 2005), flagella (Choe et al., 2022), and outer membrane proteins (Leung et al., 2019).

Environmental factors, such as temperature, are known to modulate the expression of virulence genes in fish pathogenic bacteria, allowing the bacteria to adapt to changing conditions (Guijarro et al., 2015). Previous studies have shown *E. piscicida* uses the two‐component system PhoP‐PhoQ to sense changes in environmental temperature and regulate expression of the T3SS and T6SS, with reduced expression of these secretion systems at 37°C compared to 25°C (Guijarro et al., 2015; Srinivasa Rao et al., 2004). This mechanism of temperature‐dependent virulence gene expression is especially important in the context of warming trends associated with climate change, given that outbreaks of economically important fish diseases such as edwardsiellosis and lactococcosis are often precipitated by increased water temperatures (Guijarro et al., 2015). Additionally, pathogens that switch between endothermic hosts and ectothermic hosts or environmental persistence often use temperature as a signal to control expression of virulence genes to facilitate infection and/or persistence when transitioning between ambient temperatures and endothermic host physiologic temperatures (37°C) (Kamp & Higgins, 2011; Lam et al., 2014; Scheller et al., 2021). Thus, an improved understanding of the temperature‐dependent regulation of *E. piscicida* virulence factors can provide insights into its potential pathogenicity in mammals and the impact of climate change‐mediated increased water temperatures on the virulence of this emerging pathogen.

Emerging infectious fish diseases are a major concern for global aquaculture due to the substantial economic losses associated with increased treatment costs, reduced growth due to morbidity, disease‐induced anorexia, and direct losses from mortality. Moreover, there is a considerable public health concern due to the risk of waterborne and/or foodborne disease. With reports of *E. piscicida* outbreaks steadily increasing over the past decade, with an expanding host and geographic range and a putative association with climate‐change mediated increased water temperatures, it is crucial to understand the role of temperature on the expression of *E. piscicida* virulence factors. To this end, liquid chromatography tandem mass spectrometry (LC‐MS/MS) was used to characterize the proteomic profiles of *E. piscicida* isolates from each of the six known clades grown at three experimental temperatures: 17°C, to simulate a cool‐water system; 27°C, to simulate a warm‐water system; and 37°C, to simulate a mammalian system.

## 2 MATERIALS AND METHODS

### 2.1 Bacterial isolates

Eighteen archived isolates of *E. piscicida* collected from diseased fish in the United States, representing the six identified genetic groups, were used in this study (Table 1). Isolates were revived from frozen stock by isolation streaking on trypticase soy agar supplemented with 5% sheep blood (SBA, University of California Davis, Biological Media Services, USA) and grown overnight at 28°C. Individual colonies were expanded in brain heart infusion broth (BHI; MP Biomedicals) and stored in 1 mL aliquots with 20% glycerol (v/v) to create new working stocks for each isolate, which were maintained at −80°C. Prior to each assay, frozen bacteria were grown on SBA at 28°C for 24 h. For *E. piscicida* a 0.5 McFarland standard (~1.5 × 10^8^ CFU/mL) corresponded to a bacterial suspension in phosphate‐buffered saline (PBS) with an optical density at 600 nm (OD_600_) of 0.15 (BioPhotometer Plus, Eppendorf AG). Bacterial concentration was confirmed via serial dilution and spot‐plating of 10 μL aliquots in triplicate on SBA. After 24 h incubation at 28°C, the number of colony‐forming units (CFU) on the plates was counted to estimate the CFU/mL of the bacterial suspension.

**TABLE 1:** List of *Edwardsiella piscicida* isolates used in this study.

Isolate	Clade	Source	References	NCBI accession	Genome length (bp)	%GC	CDS	rRNA	tRNA	ncRNA	Plasmid length (bp)
6‐30 IP D16‐014	Clade I	Largemouth bass	Nguyen et al. (2021)	CP150945‐CP150946	3789405	59.6	3363	28	98	5	3067
		(*Micropterus salmoides*)									
34‐68^a^	Clade I	Atlantic salmon	Nguyen et al. (2021)	CP150944	38929320	59.6	3375	28	98	5	n/a
		(*Salmo salar*)									
R1843	Clade I	Largemouth bass	Camus et al. (2019)	JBCHVA000000000^b^	3305113	59.6	3458	30	101	5	n/a
		(*M. salmoides*)									
LADL‐16005	Clade II	Barramundi	Loch et al. (2017)	CP150941	3760097	59.8	3325	28	103	5	n/a
		(*Lates calcarifer*)									
S11‐233^a^	Clade II	Channel catfish	Griffin et al. (2014)	CP150940	3802971	59.7	3372	28	99	5	n/a
		(*Ictalurus punctatus*)									
A15‐1679	Clade II	Blotched fantail stingray	Camus et al. (2016)	CP150939	3786820	59.8	3323	28	98	5	n/a
		(*Taeniura meyeni*)									
32‐97	Clade III	Atlantic salmon	Nguyen et al. (2021)	CP150938	3756321	59.9	3296	28	99	5	n/a
		(*S. salar*)									
35‐70^a^	Clade III	Atlantic salmon	Nguyen et al. (2021)	CP150937	3756329	59.9	3294	28	99	5	n/a
		(*S. salar*)									
Reds‐S19‐11‐E	Clade III	Rainbow trout	Reichley et al. (2017)	CP150936	3826819	59.7	3393	31	103	5	n/a
		(*Oncorhynchus mykiss*)									
S11‐285	Clade IV	Channel catfish	Griffin et al. (2014)	CP150934‐CP150935	3917999	59.6	3501	28	103	5	3164
		(*I. punctatus*)									
S07‐534	Clade IV	Channel catfish	Griffin et al. (2014)	CP150933	3968788	59.5	3569	28	100	5	n/a
		(*I. punctatus*)									
S12‐281^a^	Clade IV	Hybrid catfish	Griffin et al. (2014)	CP150931‐CP150932	3917264	59.6	3491	28	104	5	3164
		(♀ *I. punctatus* × ♂ *I. furcatus*)									
S16‐567	Clade V	Hybrid catfish	Griffin et al. (2019)	CP150929‐CP150930	3801030	59.7	3420	28	103	5	77653
		(♀ *I. punctatus* × ♂ *I. furcatus*)									
C1490	Clade V	Largemouth bass	Fogelson et al. (2016)	CP150927‐CP150928	3996733	59.4	3699	28	103	5	77653
		(*M. salmoides*)									
LADL99‐462^a^	Clade V	Channel catfish	Griffin et al. (2014)	CP150925‐CP150926	3816076	59.7	3356	28	102	5	3616
		(*I. punctatus*)									
S16‐278	Clade VI	Hybrid catfish	Griffin et al. (2019)	CP150923‐CP150924	3837991	59.6	3411	25	101	5	3781
		(♀ *I. punctatus* × ♂ *I. furcatus*)									
MA97‐004^a^	Clade VI	Tilapia	Griffin et al. (2014)	CP150922	3839658	59.6	3387	28	101	5	n/a
		(*Tilapia* sp.)									
D16‐038	Clade VI	Largemouth bass	Nguyen et al. (2021)	CP150920‐CP150921	3843154	59.6	3414	28	102	5	2358
		(*M. salmoides*)									

^a^ Isolates were used for in‐vitro proteomics.

^b^ Genome is not circularized.

### 2.2 Bacterial growth curves

McFarland standard suspensions of 0.5 in PBS were generated for each bacterial isolate (three representative isolates per *E. piscicida* clade) and then serially diluted to 1:1000 in BHI. One‐hundred microlitres of each dilution were plated in quintuplicate onto 96‐well plates, with six wells per plate containing 100 μL of sterile BHI as background controls. Plates were incubated for 48–72 h at one of three experimental temperatures (17, 27, or 37°C). Throughout the incubation period, the absorbance at OD_600_ of each sample was measured at 1‐h intervals using a Cytation™ 5 Imaging Reader (BioTek, USA). Growth curve measurements were repeated in duplicate for each experimental temperature, and OD measurements were adjusted based on BHI control wells.

### 2.3 Peptide preparation

One representative isolate per clade was selected based on demonstrated high morbidity and mortality in salmonids and catfish (López‐Porras et al., 2021; Nguyen et al., 2021). Individual colonies from selected isolates were suspended in 5 mL of sterile BHI and incubated to the late exponential‐phase of growth, as determined by prior growth curve experiments, at one of three experimental temperatures (48 h at 17°C, 24 h at 27°C, and 13 h at 37°C) with constant orbital shaking. The late‐exponential phase of growth was selected for proteomic profiling as many other pathogenic bacteria demonstrate upregulated expression of virulence factors, such as the T6SS, in the late‐exponential phase and early stationary phase (Hayashi et al., 2010; Hespanhol et al., 2023; Sun et al., 2017). Following incubation, samples were pelleted via centrifugation at 4500 rpm for 25 min. Bacterial proteins were extracted from all samples according to the manufacturer's suggested protocols for the B‐PER™ with Enzymes Bacterial Protein Extraction Kit (Thermo Fisher Scientific, USA), with minor modifications as described. Briefly, 10 μL/mL of Thermo Scientific™ Halt™ Protease Inhibitor Cocktail (Thermo Fisher Scientific, USA) was added to the B‐PER reagent, vortexed, and then 5 mL of the resulting solution per 1 g of bacterial pellet was combined and pipetted until homogenized. Samples were incubated at room temperature for 15 min, then concentrated by centrifugation for 5 min. The supernatant containing B‐PER‐extracted bacterial proteins was stored at 4°C. The remaining pellet was further processed following manufacturer recommendations for the Inclusion Body Solubilization (IBS) Reagent (Thermo Fisher Scientific, USA), and the collected supernatant was stored at 4°C.

Extracted bacterial proteins (one isolate per clade, three technical replicates per isolate) were submitted to the UC Davis Proteomics Core Facility for digestion and liquid chromatography tandem mass spectrometry (LC‐MS/MS). Protein concentration was quantified using the Pierce™ BCA Protein Assay Kit (Thermo Fisher Scientific, USA). Each sample (150 μg of protein) was digested on an S‐Trap™ Digestion column plate. Initially, 10 mM dithiothreitol (DTT) was added and incubated at 50°C for 10 min and rested at room temperature for 10 min. Next, 5 mM iodoacetamide (IAA) was added and incubated at room temperature for 30 min in the dark. The samples were acidified with 12% phosphoric acid, followed by the addition of freshly made S‐trap buffer (90% methanol, 100 mM TEAB, pH 7.1), and mixed immediately by inversion. The entire acidified lysate buffer mix was transferred to the S‐trap plate and pushed through with a Resolvex A200 (Tecan Group Ltd., Männedorf, Switzerland) until all the solution had passed through. Columns were washed with 400 μL of S‐trap buffer. Trypsin enzyme digest buffer was carefully added (1:25 enzyme: total protein in 120 uL 50 mM Triethyl Ammonium Bicarbonate (TEAB), pH 8.0) to the column. After 2 h of incubation at 37°C, the same amount of trypsin and TEAB were added to the S‐trap as a boost step, and the reaction continued overnight at 37°C. The following day, peptides were eluted from the S‐trap. Peptide elution steps included 80 μL of 50 mM TEAB (pH 8.0), 80 μL of 0.5% formic acid, 80 μL of the solution containing 50% acetonitrile, and 0.5% formic acid. The final pooled elution was dried down in a speed‐vacuum. Peptides were resuspended in 0.1% TFA and 2% ACN and quantified using Pierce™ Quantitative Fluorometric Peptide Assay (Thermo Fisher Scientific, USA).

### 2.4 Liquid chromatography tandem mass spectrometry

Peptides were resolved on a Dionex UltiMate 3000 RSLC system (Thermo Fisher Scientific, USA) using a PepSep analytical column (PepSep, Denmark): 150 μm x 8 cm C18 column with 1.5 μm particle size (100 Å pores), preceded by a Pepmap Neo C18 5UM guard column, and heated to 40°C. Separation was performed in a total run time of 30 min with a flow rate of 500 μL/min with mobile phases A: water/0.1% formic acid and B: 80%ACN/0.1% formic acid.

### 2.5 Mass spectrometry data independent analysis

Peptides were directly eluted onto an Orbitrap Exploris 480 instrument (Thermo Fisher Scientific, Bremen, Germany). Spray voltage was set to 1.8 kV, funnel RF level at 45, and heated capillary temperature at 275°C. Experiments full MS resolution was set to 60,000 at m/z 200 and full MS automatic gain control (AGC) target was 300% with an IT of 45 msec. The mass range was set to 350–1200. The AGC target value for fragment spectra was set at 1000%. Mass Windows of 22 Da were used with an overlap of 1 Da. The Resolution was set to 30,000 and IT to Auto. The Normalized collision energy was set at 30%. All data were acquired in profile mode using positive polarity, peptide match was set to off, and isotope exclusion was on.

### 2.6 Genome sequencing

Isolates were revived from cryostock on Mueller Hinton II agar plates supplemented with 5% defibrinated sheep blood and incubated at 37°C for 24 h. Single colonies were then inoculated in porcine brain heart infusion broth (Becton, Dickinson and Company, Franklin Lakes, NJ) and incubated at 28°C for 18 h with shaking (200 rpm). A 1.5 mL aliquot was concentrated by centrifugation (17,000× *g* for 3 min) and genomic DNA isolated using the PuroSPIN™ Genomic DNA Purification Plus Kit (Luna Nanotech, Ontario, Canada). Libraries were prepared using the Oxford Nanopore Native Barcoding Kit 24 V14 (SQK‐NBD114.24; Oxford Nanopore Technologies, Oxford, United Kingdom) and sequenced on a GridIon using R10.4.1 flow cells. Basecalling was performed with the super‐accurate model in guppy (v3.5.1). The resulting data were filtered for a minimum length of 1000 bp and a minimum quality score of 10 with NanoFilt (v2.8.0) (De Coster et al., 2018) prior to being assembled with Canu (v2.2) (Koren et al., 2017) and corrected with Medaka (v1.8.0) (Oxford Nanopore Technologies Ltd., 2018).

### 2.7 Data analysis

Statistical analyses for bacterial growth curves were performed in GraphPad Prism v10.0.1. The area under the curve (AUC) was calculated for each growth curve using the mean OD_600_ from the negative control wells as the baseline and compared across bacterial clades and growth temperatures using Brown‐Forsythe and Welch ANOVA and Tukey's multiple comparisons test. All growth curves were fit to a nonlinear regression model to allow for calculation of peak OD_600_, maximal growth rate, and lag time. Temperature‐dependent and interclade differences in these growth curve parameters were analysed using Ordinary one‐way ANOVA tests and Tukey's multiple comparisons tests. A value of *p* < .05 was considered significant for all statistical analyses.

Mass spectrometry data were analysed using Spectronaut 17.5 (Biognosys Schlieren, Switzerland) using the directDIA workflow with the default settings. Briefly, trypsin/P Specific was set for the enzyme, allowing two missed cleavages. Fixed modifications were set for carbamidomethyl, and variable modification were set to acetyl (protein N‐term) and oxidation. For DIA search identification, PSM and Protein Group FDR were set at 0.01%. A minimum of 2 peptides per protein group were required for quantification. Analyses of proteomic data were performed in Spectronaut® (Biognosys Inc, USA), and peptides were compared to the *E. piscicida* Uniprot/TrEMBL database for protein identification. Precursor q‐value cutoff was set to 0.01, precursor posterior error probability (PEP) cutoff was set to 0.2, and protein PEP cutoff was set to 0.75. Principal component analysis (PCA) was performed based on the protein group quantity of the protein profiles for each sample in Spectronaut. Proteins with a *p*‐value of < .05, as determined by unpaired Student's *t*‐test, and an absolute log2 ratio ≥1 were considered differentially expressed. Virulence factors and adaptive response proteins were identified by comparison with the “Virulence Factors of Pathogenic Bacteria Database” (sequence identity >80%, e‐value ≤1e^−3^) (VFDB, http://www.mgc.ac.cn/VFs/, accessed on 1 November 2023) and with previously reported data based on predicted or demonstrated virulence function in Edwardsiella sp. and other Gram negative pathogens (Table S2).

## 3 RESULTS

### 3.1 Growth curve patterns consistently differed based on temperature but not clade

**FIGURE 1:**
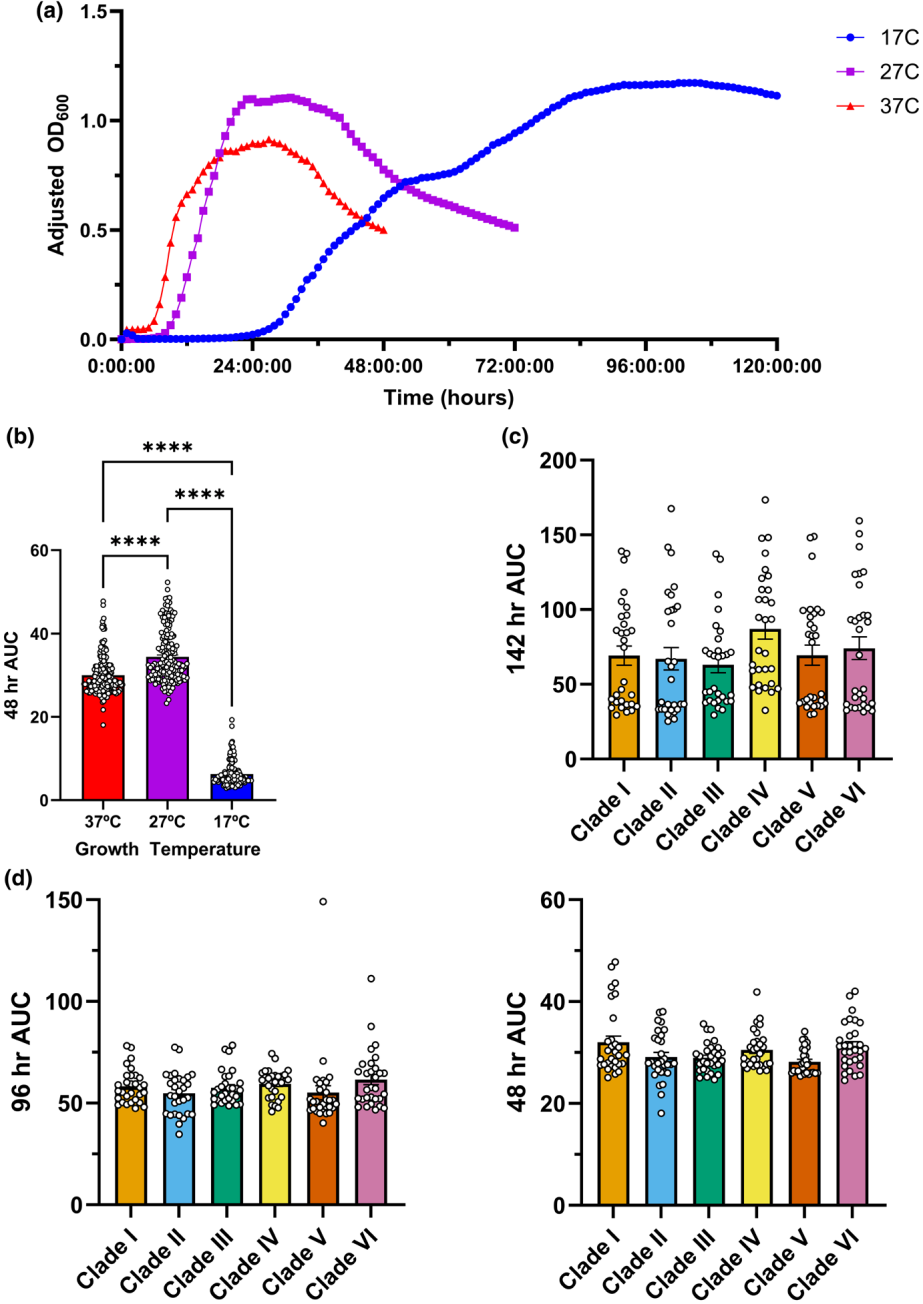
Growth curves for eighteen selected *E. piscicida* isolates (three isolates per clade) grown at 17, 27, and 37°C in Brain Heart Infusion (BHI) broth. Each isolate was run in quintuplicate per experiment, and each experiment was repeated twice. (a) Temperature‐dependent growth curve patterns for *E. piscicida*. Each data point represents the mean adjusted Optical Density (OD)_600_ at that time point. (b) Temperature‐dependent differences in 48 ‐h Area Under the Curve (AUC). (c) Clade‐dependent AUC for *E. piscicida* at 17°C. (d) Clade‐dependent AUC for *E. piscicida* at 27°C. (e) Clade‐dependent AUC for *E. piscicida* at 37°C. Asterisks indicate statistical significance between groups as determined by One‐Way ANOVA with Tukey’s multiple comparisons tests: *p < 0.05; **p < 0.01; ***p < 0.001; ****p < 0.0001.

All *E. piscicida* isolates were successfully able to grow and replicate at all three experimental growth temperatures. *Edwardsiella piscicida* presented significant temperature‐dependent differences in growth curve patterns in BHI (Figure 1). Isolates grown at 17°C had significantly higher (*p* < .05) mean peak OD_600_ (1.175 AU) and extended mean lag time (24.22 h) than isolates grown at 27°C (1.064 AU; 5.343 h) or 37°C (0.9058 AU; 3.271 h). Isolates grown at 37°C had the lowest mean peak OD_600_ (*p* < .05) of the three growth temperatures. Isolates grown at 27°C had the fastest mean maximal growth rate (*p* < .05; 0.03281 AU/h), whilst isolates grown at 17°C had the slowest mean maximal growth rate (*p* < .05; 0.01107 AU/h). There was no significant interclade difference in mean peak OD_600_ for any of the three experimental temperatures. When grown at 17°C, Clade IV *E. piscicida* isolates had the highest mean maximal growth rate of the six bacterial clades (*p* < .05); however, this trend was not present when bacteria were grown at 27°C or 37°C, which demonstrated no significant interclade differences in mean maximal growth rate. Additionally, at 17°C, clade II isolates had a significantly extended lag time (*p* < .05) compared to clade IV and V isolates, but there was no similar trend at 27°C or 37°C for which there were no significant differences in mean lag time.

All *E. piscicida* isolates grown had significantly different (*p* < .05) 48 h AUCs among the three growth temperatures (mean AUC 6.289 at 17°C, mean AUC 34.41 at 27°C, and mean AUC 30.02 at 37°C). There was no significant inter‐clade difference in AUC at any of the three experimental temperatures.

### 3.2 *Edwardsiella piscicida* displayed temperature‐dependent differences in virulence factor abundance

**FIGURE 2:**
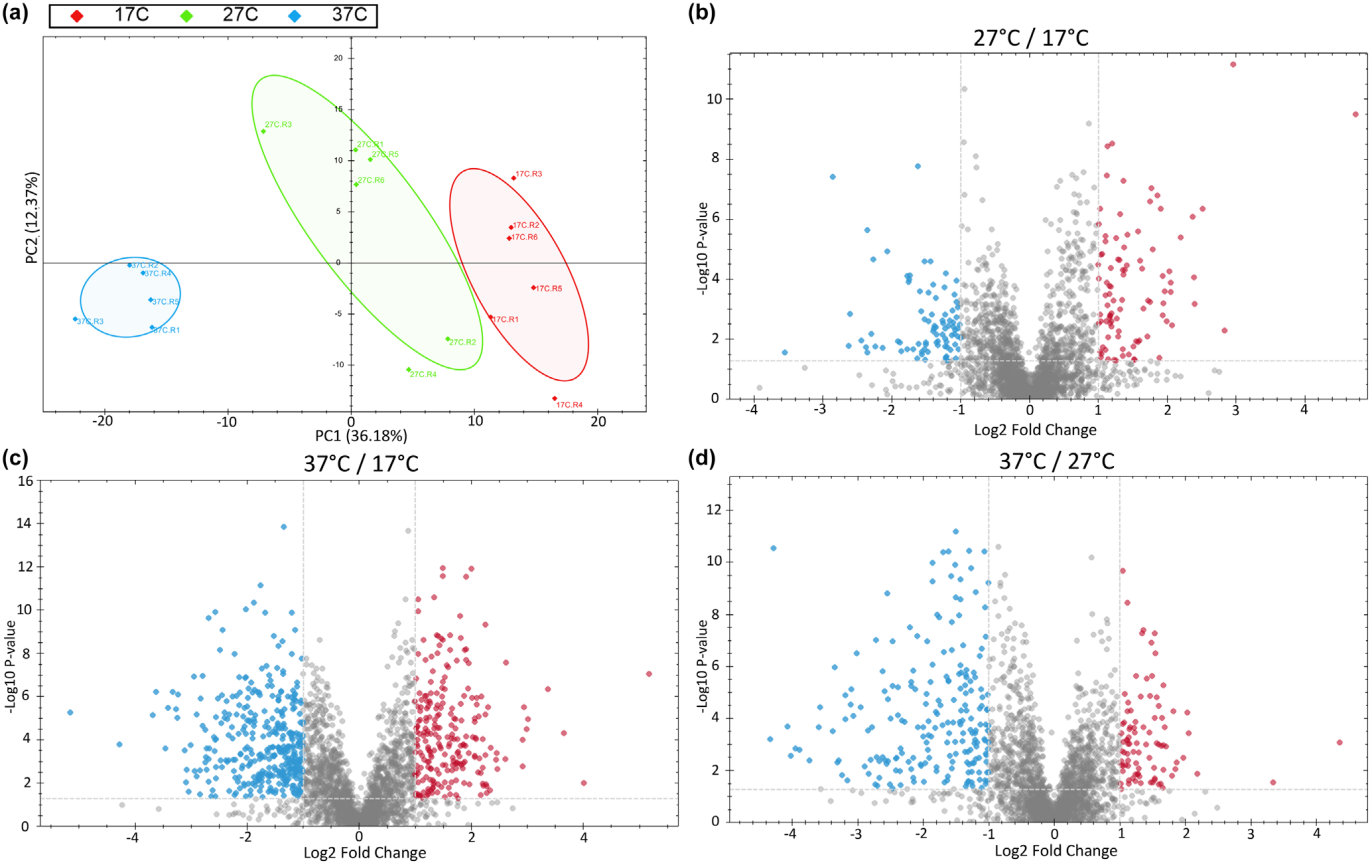
Temperature‐dependent in vitro proteomic profile of *Edwardsiella piscicida*. (a) Principal Component Analysis (PCA) plot displaying the sample protein profile clustering of the three experimental temperature groups. Volcano plots depicting differential abundance of proteins based on growth temperature (Group 1/Group 2): (b) 27°C/17°C; (c) 37°C/17°C; (d) 37°C/27°C. Proteins with significantly increased relative abundance (Log2 ratio ≥1, *p* < .05) are depicted in red, and proteins with significantly decreased relative abundance (Log2 ratio ≤ −1, *p* < .05) are depicted in blue.

**FIGURE 3:**
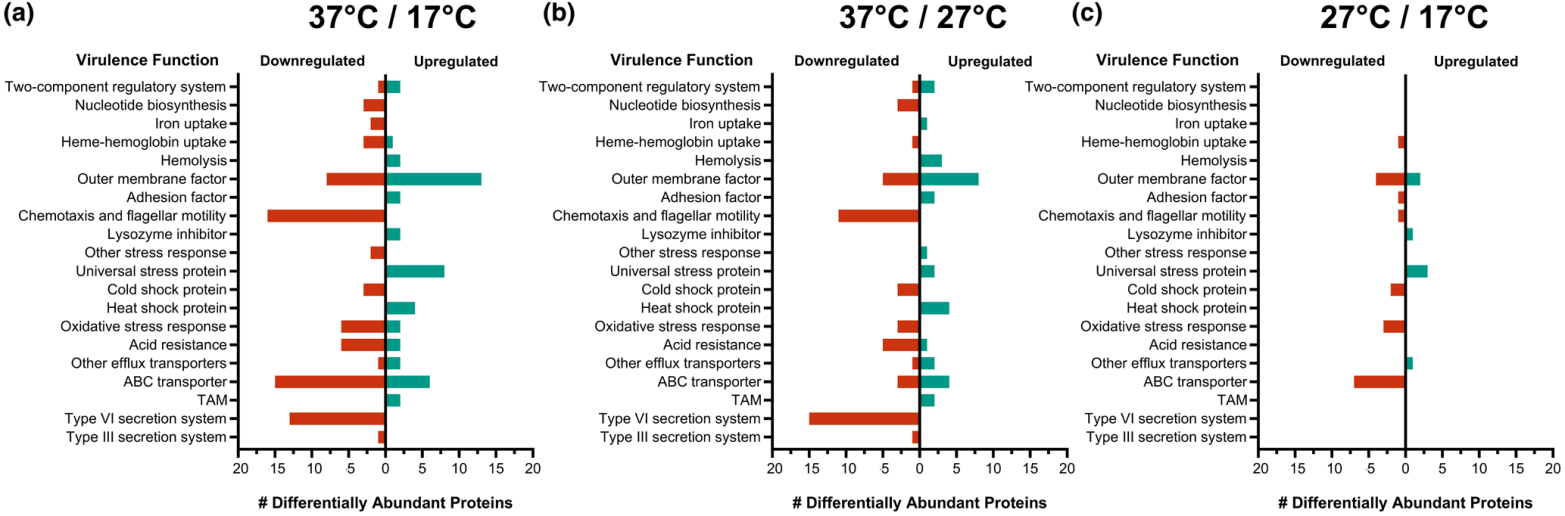
Summary of differential *E. piscicida* virulence factor regulation based on in‐vitro growth temperature (Group 1/Group 2): (a) 37°C/17°C; (b) 37°C/27°C; (c) 27°C/17°C. Upregulated proteins for each comparison were defined as those with a log2 ratio ≥1 and *p* < .05 (depicted in green). Downregulated proteins for each comparison were defined as those with a log2 ratio ≤ −1 and *p* < .05 (depicted in red).

A total of 26,997 unique peptides were identified, which corresponded to 2739 proteins from the *E. piscicida* Uniprot/TrEMBL database. When comparing across bacterial clades, we found no significant or consistent patterns in differential protein abundance. Protein profiles did, however, differ between temperatures, with distinct clusters visible upon principal component analysis (Figure 2). When evaluating protein regulation based on bacterial growth temperature, the greatest number of differentially abundant proteins (absolute log2 ratio ≥1; *p* < .05) was identified when comparing isolates grown at 37°C to isolates grown at 17°C (595 differentially abundant proteins) (Figure 2; Table S1). Comparing isolates grown at 37°C to isolates grown at 27°C identified 298 differentially abundant proteins, whilst the least number of differentially abundant proteins (180) was identified when comparing isolates grown at 27°C to isolates grown at 17°C. Of the 1074 comparisons that identified significantly differentially abundant proteins, 150 were identified as virulence factors and adaptive response proteins (Figure 3; Table S2) and included proteins such as ATP‐binding cassette (ABC), flagellin, and Type VI secretion system effectors. Both structural and effector T6SS proteins were downregulated at 37°C compared to 27°C and 17°C. Additionally, proteins associated with chemotaxis, flagellar motility, nucleotide biosynthesis, oxidative stress response, and acid resistance were downregulated at 37°C compared to 27°C and 17°C. Conversely, at 37°C, *E. piscicida* upregulated production of proteins associated with adhesion, haemolysis, and the translocation and assembly module (TAM). The proteomic profiles of several crucial outer membrane proteins differed depending on the bacterial growth temperature, with both OmpA and OmpW displaying increased abundance with increasing growth temperature. Unsurprisingly, cold shock proteins were upregulated at 27°C and 17°C, compared to 37°C, whilst heat shock proteins and universal stress proteins were upregulated at 37°C.

## 4 DISCUSSION

Results indicate growth of *E. piscicida* at cooler temperatures (17°C) is slower, with an extended lag phase and reduced maximal growth rate than at higher temperatures. Conversely, although isolates grown at 37°C demonstrated a faster growth rate than isolates at 17°C, they had a reduced maximal OD_600_ and were quicker to enter the death phase of bacterial growth than the other two experimental temperatures. These results concur with previous findings of an optimum growth temperature for *E. piscicida* around 28 and 30°C, as isolates in the current study grown at 27°C displayed the fastest maximal growth rate of any of the three experimental temperatures, had a shorter lag phase than isolates grown at 17°C, and had a peak OD_600_ equivocal to isolates grown at 17°C (Abayneh et al., 2013; Griffin et al., 2013). Thus, overall growth of *E. piscicida* appeared to be reduced and/or limited at cooler water temperatures (17°C) or mammalian physiologic temperatures (37°C) when compared to warmer water temperatures (27°C). Interestingly, no consistent interclade differences in bacterial growth patterns were observed.

Proteomic analysis identified significant alterations in the regulation of several crucial virulence factors between *E. piscicida* isolates grown at mammalian temperatures (37°C) and isolates grown at cool‐water (17°C) and warm‐water (27°C) temperatures. Many vital virulence mechanisms, including the T6SS, chemotaxis, flagellar motility, oxidative stress response, and acid resistance response, were downregulated at 37°C compared to 27°C and 17°C. The T6SS of *E. piscicida* is comprised of multiple structural proteins forming a contractile injection apparatus that facilitates transport of effectors directly into target host cells for bacterial adhesion, invasion, and intracellular replication (Li et al., 2021). Functional T6SS plays a pivotal role during in vivo *E. piscicida* infection and is essential for full virulence of *E. piscicida* (Hu et al., 2019). These results showed downregulation of both structural and effector T6SS proteins at mammalian temperatures compared to warm or cold‐water temperatures, which is consistent with previous findings of decreased T6SS protein secretion and effector gene expression at 37°C compared to 25°C due to temperature‐dependent transcriptional control by the two‐component system PhoP‐PhoQ (Guijarro et al., 2015). This finding suggests that *E. piscicida* may have decreased virulence in a mammalian system due to impaired T6SS‐mediated invasion into and replication within host macrophages. Furthermore, survival within macrophages by *E. piscicida* may be further impaired by temperature‐dependent downregulation of oxidative stress and acid resistance proteins at mammalian physiological temperatures. Interestingly, this study only identified one T3SS protein, CesD/SycD/LcrH family type III secretion system chaperone, with altered temperature‐dependent regulation in contrast to previous studies, which observed decreased expression of T3SS effector proteins at 37°C compared to 25°C (Srinivasa Rao et al., 2004). In contrast to previous studies (Srinivasa Rao et al., 2004), all other *E. piscicida* T3SS effector and structural proteins detected in this study demonstrated similar abundance across the three experimental growth temperatures in the presented study (Table S3). Additionally, several of these T3SS proteins, such as EsaG and EsaG, were detected in relatively lower abundances compared to the average protein quantity and were inconsistently detected across samples. One possible explanation for this finding is that different culture conditions could impact the presence of signals necessary to activate transcription and translation of T3SS effectors. As bacteria in this experiment were grown in extracellular growth medium conditions, there may be intracellular signals that would significantly alter the production of T3SS proteins in vivo (Zhang et al., 2023).

Major chemotaxis proteins (CheY, CheZ, CheW, and CheA) and flagellar proteins (flagellin, FliN, and FliM) were downregulated at 37°C compared to 27 or 17°C. This finding could suggest decreased virulence at mammalian temperatures due to impaired chemotactic, motility, and invasion capabilities. This is consistent with reports by Griffin et al. (2020), which indicated limited pathogenesis for *E. piscicida* in a murine model. Alternatively, other opportunistic environmental bacterial pathogens are known to utilize temperature as a signal to control expression of flagellar motility genes when transitioning between ambient temperature environments and warm‐blooded hosts. *Listeria monocytogenes* is a foodborne/waterborne agent of gastrointestinal illness that utilizes its flagella for motility and persistence in the extracellular environment and suppresses flagellar motility genes after infection in response to mammalian physiologic temperature in order to facilitate full virulence (Kamp & Higgins, 2011). Additionally, many Gram negative opportunistic pathogens are known to utilize environmental signals such as temperature to trigger a switch from a motile, planktonic state to a sessile, biofilm state either to facilitate host cell colonization or as a survival response to stressors (Rossi et al., 2018). The concurrent downregulation of flagellar motility and chemotactic proteins and upregulation of adhesion factors and universal stress proteins identified in this study could indicate a transition by *E. piscicida* from a motile form at cool‐water or warm‐water temperatures to a biofilm mode at 37°C as is seen in marine *Bacillus* species in which biofilm formation is enhanced at 37°C to facilitate survival and environmental persistence (Rajitha et al., 2021), or could suggest a host adaptative response for enhanced adhesion to mucosal surfaces and evasion of host immune defences as is seen in other food and water‐borne enteric pathogens such as *Yersinia enterocolitica* and *Escherichia coli* (Konkel & Tilly, 2000; Rudenko et al., 2019). This second scenario is supported by the current study, with upregulation of OmpW at mammalian physiologic temperatures, consistent with previous studies linking OmpW to adaptive responses in *E. coli* when transitioning from the environment to a warm‐blooded host (Brambilla et al., 2014). Further investigation into the in vitro and/or in vivo impact of these temperature‐dependent alterations in virulence factor expression on *E. piscicida* pathogenicity is warranted to better understand the role of temperature in the pathogenesis of piscine edwardsiellosis and to further characterize the potential zoonotic risk of *E. piscicida*.

## AUTHOR CONTRIBUTIONS

**Kim L. Jacobsen:** Formal analysis; visualization; writing – original draft; methodology; investigation. **Matt Griffin:** Conceptualization; data curation; methodology; investigation; supervision; project administration; writing – review and editing; funding acquisition; validation. **Brett S. Phinney:** Formal analysis; methodology; investigation; writing – review and editing. **Michelle Salemi:** Formal analysis; methodology; investigation; writing – review and editing. **Zeinab Yazdi:** Formal analysis; investigation; writing – review and editing. **Sujita Balami:** Investigation; methodology; formal analysis; writing – review and editing. **Caitlin E. Older:** Data curation; formal analysis; visualization; writing – review and editing; investigation; methodology; supervision; software; validation. **Esteban Soto:** Conceptualization; formal analysis; supervision; investigation; methodology; writing – review and editing; project administration; funding acquisition; resources.

## FUNDING INFORMATION

This work was funded by a grant from the Foundation for Food & Agriculture Research (FFAR).

## CONFLICT OF INTEREST STATEMENT

The authors declare no conflicts of interest.

## Supporting information


**Table S1.** Summary of differentially abundant proteins based on growth temperature as determined by LC‐MS/MS.

**Table S2.** Summary of differentially abundant virulence factors based on growth temperature as determined by LC‐MS/MS.

**Table S3.** Quantities of characterized proteins for all samples as determined by LC‐MS/MS.

## Data Availability

The data presented in this study are available on request. Bacterial genomes are available from NCBI via accession numbers listed in Table 1.
